# Plant Defense Responses to Biotic Stress and Its Interplay With Fluctuating Dark/Light Conditions

**DOI:** 10.3389/fpls.2021.631810

**Published:** 2021-03-04

**Authors:** Zahra Iqbal, Mohammed Shariq Iqbal, Abeer Hashem, Elsayed Fathi Abd_Allah, Mohammad Israil Ansari

**Affiliations:** ^1^Molecular Crop Research Unit, Department of Biochemistry, Chulalongkorn University, Bangkok, Thailand; ^2^Amity Institute of Biotechnology, Amity University, Lucknow, India; ^3^Botany and Microbiology Department, College of Science, King Saud University, Riyadh, Saudi Arabia; ^4^Mycology and Plant Disease Survey Department, Plant Pathology Research Institute, ARC, Giza, Egypt; ^5^Plant Production Department, College of Food and Agricultural Sciences, King Saud University, Riyadh, Saudi Arabia; ^6^Department of Botany, University of Lucknow, Lucknow, India

**Keywords:** biotic stress, dark, defense response, light, plant protection, transcription factor

## Abstract

Plants are subjected to a plethora of environmental cues that cause extreme losses to crop productivity. Due to fluctuating environmental conditions, plants encounter difficulties in attaining full genetic potential for growth and reproduction. One such environmental condition is the recurrent attack on plants by herbivores and microbial pathogens. To surmount such attacks, plants have developed a complex array of defense mechanisms. The defense mechanism can be either preformed, where toxic secondary metabolites are stored; or can be inducible, where defense is activated upon detection of an attack. Plants sense biotic stress conditions, activate the regulatory or transcriptional machinery, and eventually generate an appropriate response. Plant defense against pathogen attack is well understood, but the interplay and impact of different signals to generate defense responses against biotic stress still remain elusive. The impact of light and dark signals on biotic stress response is one such area to comprehend. Light and dark alterations not only regulate defense mechanisms impacting plant development and biochemistry but also bestow resistance against invading pathogens. The interaction between plant defense and dark/light environment activates a signaling cascade. This signaling cascade acts as a connecting link between perception of biotic stress, dark/light environment, and generation of an appropriate physiological or biochemical response. The present review highlights molecular responses arising from dark/light fluctuations *vis-à-vis* elicitation of defense mechanisms in plants.

## Introduction

Plants are prone to a number of biotic stress conditions. The suite of molecular and cellular processes is triggered once the plant senses stress (Rejeb et al., 2014; Lamers et al., 2020), which in turn activates a cross-wired mesh of morphological, physiological, and biochemical mechanisms (Nejat and Mantri, 2017; Saijo and Loo, 2020). Plants have developed complex sensory mechanisms to identify biotic invasion and overcome the detriment of growth, yield, and survival (Rizhsky et al., 2004; Lamers et al., 2020). Consequently, plants have evolved a surfeit of responses to defend themselves against attacks by a broad spectrum of pests and pathogens, including viruses, nematodes, bacteria, fungi, and herbivorous insects (Hammond-Kosack and Jones, 2000). Thus, plants tend to strike a balance between their response and biotic stress to combat the deleterious effect on their survival (Peck and Mittler, 2020). The molecular mechanisms contributing toward plant defense responses had been elucidated to a great depth (Cheng et al., 2012; Wang Z. et al., 2019). But how and why different signaling pathways converge to biotic stress responses still remain obscure. The light signaling pathway is one such area of interest amongst the research community.

Dark and light alterations are fundamental to plant survival. It affects all aspects of plant growth and development. The light signals are perceived by photoreceptors, which are capable of discriminating various wavelengths of light (Franklin et al., 2004). Photoreceptors, namely, phytochromes (sense red and far-red light), phototropins, and cryptochromes (sense blue light and UV light), develop cues from qualitative and quantitative light alterations (Christie, 2007; Yu et al., 2010; Tilbrook et al., 2013). This sensing activates several signal transduction pathways, which in turn regulate plant growth, physiology, morphology, and immunity (Kami et al., 2010; Moreno and Ballaré, 2014; Mawphlang and Kharshiing, 2017; Tripathi et al., 2019). In addition, photosynthetic reactions themselves regulate biochemical machinery in plant tissues (Lu and Yao, 2018). This is evident by the point that a number of genes are transcriptionally induced by the circadian clock in *Arabidopsis thaliana* and other plants (Harmer et al., 2000; Creux and Harmer, 2019). Circadian clock has been reported to meticulously regulate the defense machinery in plants (Sharma and Bhatt, 2015).

There are two developmental fates of seedling upon germination that are primarily dependent upon the presence or absence of light. In the presence of light, seedlings develop a shorter hypocotyl and open green cotyledons. This default pathway of plant development is termed photomorphogenesis (Bae and Choi, 2008; Pham et al., 2018). On the contrary, plants grown in dark conditions undergoes skotomorphogenesis (plant development under dark conditions), allocating the resources toward hypocotyl elongation rather than on cotyledon or root development (Josse and Halliday, 2008). Elongated hypocotyls, closed cotyledons, and an apical hook at the shoot meristem are characteristic to skotomorphogenetic plant development (Pham et al., 2018). Skotomorphogenesis is accomplished by repressing genes implicated in de-etiolation and photomorphogenic development (Josse and Halliday, 2008). Additionally, the effect of dark/light alteration is not only limited to plant growth and development, but it also impacts other responses to the environment such as defense against pests and pathogens (Ballaré, 2014). Extensive research exists to vindicate the effect of dark/light alterations on plant defense responses, extending from biological to ecological scales (Huner et al., 1998; Roberts and Paul, 2006; Kazan and Manners, 2011; Ballare et al., 2012; Kangasjärvi et al., 2012; Hua, 2013; Garcia-Guzman and Heil, 2014; Saijo and Loo, 2020). But the in-depth mechanistic details with regard to the complex regulatory networks are yet to be explored. The basic research in this direction can assist the idea of sustainable agriculture to ensure food security for the ever-growing world population (Sánchez-Muros et al., 2014; González de Molina et al., 2017; Iqbal et al., 2020a; Saiz-Rubio and Rovira-Más, 2020). The present review recapitulates biochemical, physiological, and molecular aspects of biotic stress and plant defense responses operating in light/dark scenarios.

## Biotic Stress and Plant Defense Responses

A number of pests, parasites, and pathogens are responsible for infecting plants and inciting biotic stress. Fungal parasites can be either necrotrophic (kill host cell by toxin secretion) or biotrophic (feed on living host cell). They are capable of inducing vascular wilts, leaf spots, and cankers in plants (Laluk and Mengiste, 2010; Doughari, 2015; Sobiczewski et al., 2017). Nematodes feed on plant parts and primarily cause soil-borne diseases leading to nutrient deficiency, stunted growth, and wilting (Lambert and Bekal, 2002; Bernard et al., 2017; Osman et al., 2020). Similarly, viruses are also capable of local and systemic damage resulting in chlorosis and stunting (Pallas and García, 2011). On the contrary, mites and insects impair plants by either feeding (piercing and sucking) on them or laying eggs. The insects might also act as carriers of other viruses and bacteria (Schumann and D’Arcy, 2006). Plants have developed an elaborate immune system to combat such stresses (Taiz and Zeiger, 2006; Saijo and Loo, 2020). Plants have a passive first line of defense, which includes physical barriers such as cuticles, wax, and trichomes to avert pathogens and insects. Plants are also capable of producing chemical compounds to defend themselves from infecting pathogens (Taiz and Zeiger, 2006) (discussed in section “Effect of Dark/Light on Plant–Pathogen Interaction and Associated Mechanisms”). Additionally, plants trigger defense against biotic agents by two levels of pathogen recognition (Dangl and McDowell, 2006).

The first level of pathogen recognition encompasses pattern recognition receptors (PRRs), which identify pathogen-associated molecular patterns (PAMPs). Such plant immunity is categorized as PAMP-triggered immunity (PTI) (Monaghan and Zipfel, 2012). Phytophagous pests respond by identification of herbivore-associated elicitors (HAEs), herbivore-associated molecular patterns (HAMPs), or PRR herbivore effectors (Santamaria et al., 2013). The second level of pathogen recognition encircles plant resistance (R) proteins, which identify specific receptors from a pathogen (Avr proteins) (Dangl and McDowell, 2006; Gouveia et al., 2017; Abdul Malik et al., 2020). It is considered an effective mechanism of plant resistance to pests and involves effector-triggered immunity (ETI) (Kaloshian, 2004; Mur et al., 2008; Spoel and Dong, 2012). ETI stimulates hypersensitive responses (HRs) and triggers programmed cell death (PCD) in infected and surrounding cells (Mur et al., 2008). The proteins encoded by a majority of *R* genes have a specific domain with conserved nucleotide-binding site (NBS). The second next important domain is leucine-rich repeat (LRR). Pathogen effectors are recognized directly (physical association) or indirectly (association of an accessory protein) by NB-LRR receptors (Dodds and Rathjen, 2010). Sometimes, *R* gene-mediated plant response toward invading pathogen provokes a higher degree of defense, termed as systemic acquired resistance (SAR). SAR generates whole-plant systemic resistance against a broad spectrum of pathogens. In SAR, a local encounter results in the stimulation of resistance to the other plant organs through intraplant communication (Fu and Dong, 2013). Generally, both categories of plant immune responses induce the same reaction, but ETI is considered more rigorous to pathogen infection (Tao et al., 2003).

**FIGURE 1:**
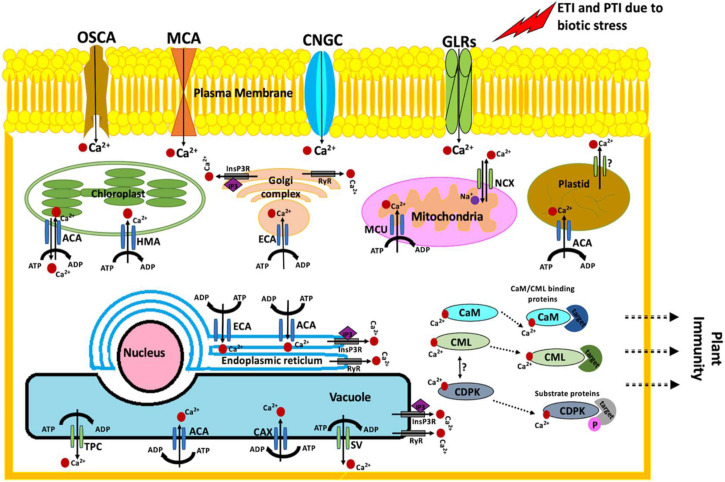
Schematic representation of biotic stress perception and Ca^2+^ signaling for the regulation of plant immune responses. The cytosolic Ca^2+^ levels increase (>200 nM) upon perceiving biotic stress: calcium (Ca^2+^), Ca^2+^–dependent protein kinases (CDPKs), calmodulin (CaM), calmodulin-like protein (CML), autoinhibited Ca^2+^-ATPases (ACAs), ER-type Ca^2+^-ATPases (ECAs), mitochondrial Ca^2+^ uniporter (MCU), P1-ATPases (e.g., HMA1), Ca^2+^ exchangers (CAX), two-pore channel (TPC), cyclic nucleotide-gated channels (CNGCs), glutamate receptor-like channels (GLRs), stretch-activated Ca^2+^ channels (OSCAs), MID1-complementing activity (MCA), phosphate (P), adenosine triphosphate (ATP), adenosine diphosphate (ADP), 1,4,5-trisphosphate receptor-like channel (InsP_3_R), 1,4,5-trisphosphate (IP_3_), cyclic ADP-ribose (cADPR)-activator ryanodine receptor-like channel (RyR), slow-activating vacuolar channel (SV), and sodium-calcium exchanger (NCX).

Perturbations in cytosolic calcium (Ca^2+^) concentrations are the earliest signaling events occurring upon the exposure of plants to biotic stress. Ca^2+^ signals are the center to plant immune signaling pathways (Seybold et al., 2014; Aldon et al., 2018). Rapid and transient perturbations in Ca^2+^ concentrations are crucial to gene reprogramming required to generate an adequate response (Reddy et al., 2011). The plant immune responses differ in their Ca^2+^ signatures. For example, Ca^2+^ transients upon PTI activation returns to basic levels within a few minutes (Lecourieux et al., 2005), while ETI involves a prolonged increase in cytosolic Ca^2+^ levels lasting for several hours (Grant et al., 2000). Lanthanum, a known Ca^2+^ channel blocker, is reported to hinder the immune responses associated with both PTI and ETI (Grant et al., 2000; Boudsocq et al., 2010). Precisely, in response to the biotic invasion, PTI and ETI activate the Ca^2+^ ion channels, resulting in an increase of cytoplasmic Ca^2+^ concentrations (Figure 1). In *A. thaliana*, cyclic nucleotide-gated channels (CNGCs), glutamate receptor-like channels (GLRs), stretch-activated Ca^2+^ channels (OSCAs), and the MID1-complementing activity (MCA) families are the four main plasma membrane Ca^2+^-permeable channels (Dodd et al., 2010; Yuan et al., 2014; Liu et al., 2018). Twenty distinct members of the CNGC family of plasma membrane Ca^2+^-permeable channels have been identified in *A. thaliana* (Meena and Vadassery, 2015; DeFalco et al., 2016). CNGCs are extensively linked to plant development and biotic stress responses (Meena and Vadassery, 2015; DeFalco et al., 2016; Breeze, 2019). In response to fungal and bacterial pathogens, the Ca^2+^-permeable channels CNGC2, CNGC4, CNGC11, and CNGC12 are reported to play critical roles in the entry of Ca^2+^ ions inside the plant cell (Yoshioka et al., 2001; Ahn, 2007). The role of CNGC2, CNGC4 (Ma et al., 2012; Chin et al., 2013), CNGC11, and CNGC12 (Yoshioka et al., 2006; Moeder et al., 2011) has been well established in plant immune responses. Very recently, the function of CNGC19 Ca^2+^ channel was also extended to herbivory-induced Ca^2+^ flux, plant defense responses against pathogen *Spodoptera litura* (Meena et al., 2019), and basal defense signaling to regulate colonization of *Piriformospora indica* on *A. thaliana* roots (Jogawat et al., 2020). The first CNGC from plants was identified nearly two decades ago in barley as a calmodulin (CaM)-binding protein (Schuurink et al., 1998). CNGCs from plants and animals are reported to possess one or more CaM-binding domains at their cytosolic N- and C-termini, but the gating of CNGCs from plants is not well deduced (DeFalco et al., 2016; Fischer et al., 2017; James and Zagotta, 2018). The progress of plant CNGC research has been relatively low due to the difficulties in electrophysiological studies encircling CNGCs. However, the recent technological advances and reliability on reverse genetics using *cngc* mutants have resulted in few successful studies (Gao et al., 2016; Chiasson et al., 2017; Wang et al., 2017; Zhang et al., 2017). CNGC7, CNGC8, and CNGC18 have been specifically reported to act together with CaM2 as a molecular switch that operates in response to cellular Ca^2+^ concentrations (Pan et al., 2019). Additionally, CNGC18 is co-expressed with CPK32, indicating the regulation of its activity by phosphorylation (Zhou et al., 2014). Similarly, GLRs, which are systematically classified into three clades—clade I (GLRs 1.1–1.4), clade II (GLRs 2.1–2.9), and clade III (GLRs 3.1–3.7) (Lacombe et al., 2001)—are linked to plant defense against *Botrytis cinerea* (Sun et al., 2019) and *Hyaloperonospora arabidopsidis* (Manzoor et al., 2013). As such, the role of *AtGLR3.3* and *AtGLR3.6* in aphid-elicited cytosolic Ca^2+^ elevation is also well established (Vincent et al., 2017). *In-vitro* kinase assay confirmed that *AtGLR3.7* is phosphorylated by CDPK3, CDPK16, and CDPK34 at serine-860 site (Wang P.-H. et al., 2019). CDPKs have been extensively associated with plant stress management and development (Singh et al., 2017). The other plasma membrane localized Ca^2+^-permeable channels, namely, OSCAs (phosphorylation of OSCA1.3 by BIK1) and MCAs (MCA1 and MCA2), are reported to regulate plant stomatal immunity (Thor et al., 2020) and manage hypergravity in *A. thaliana* hypocotyls under dark conditions, respectively (Hattori et al., 2020). Apart from the Ca^2+^ channels localized in the plasma membrane, several other Ca^2+^ channels are known to exist in the endoplasmic reticulum, mitochondria, golgi body, and plant vacuole (Singh et al., 2014; Xu et al., 2015a; Costa et al., 2018; Pandey and Sanyal, 2021). For example, autoinhibited Ca^2+^-ATPases (ACAs), ER-type Ca^2+^-ATPases (ECAs), mitochondrial Ca^2+^ uniporter (MCU), P1-ATPases (e.g., HMA1), Ca^2+^ exchangers (CAX), two-pore channel (TPC), 1,4,5-trisphosphate receptor-like channel (InsP_3_R), 1,4,5-trisphosphate (IP_3_), cyclic ADP-ribose (cADPR)-activator ryanodine receptor-like channel (RyR), slow-activating vacuolar channel (SV), and sodium-calcium exchanger (NCX) represents the organellar Ca^2+^ machinery (Figure 1). Many of these channels are reported to play pivotal roles in plant immunity (Bose et al., 2011; Pittman, 2011; Spalding and Harper, 2011; Kiep et al., 2015; Costa et al., 2017; Teardo et al., 2017; Yang et al., 2017; Demidchik et al., 2018; Taneja and Upadhyay, 2018; Pandey and Sanyal, 2021).

Once the Ca^2+^ ion enters the cell, it is sensed by an array of Ca^2+^-binding proteins. The Ca^2+^-binding proteins work as Ca^2+^ sensors decoding complex Ca^2+^ signatures (Kudla et al., 2018). Ca^2+^ sensors are highly conserved proteins and are classified into (a) CaM and CaM-like proteins (CMLs), (b) calcineurin-B-like proteins (CBLs), and (c) Ca^2+^-dependent protein kinases (CPKs) and Ca^2+^ and Ca^2+^/CaM-dependent protein kinase (CCaMK) (Cheng et al., 2002; Luan, 2009; Bender and Snedden, 2013; Ranty et al., 2016). CaM, CMLs, CBLs, and CPKs are comprehensively involved in the cross-talk of various biotic and abiotic stress signals (Ranty et al., 2016; Aldon et al., 2018). Many Ca^2+^ and Ca^2+^ sensor-associated transcription factors (TFs) are implicated in stress signaling in plants (Carrion et al., 1999; Singh and Virdi, 2013; Ranty et al., 2016; Chung et al., 2020; Shen et al., 2020). The largest and best characterized family of Ca^2+^/CaM-dependent TFs are CAMTAs (Iqbal et al., 2020b). CAMTA3 has been reported enormously as a suppressor of plant biotic defense responses (Benn et al., 2016; Jacob et al., 2018; Kim et al., 2020). It works downstream to MAP kinase (Bjornson et al., 2014) and is directly phosphorylated and degraded by flg22-responsive mitogen-activated protein kinases (MAPKs) (Jiang et al., 2020). Precisely, MPK3 and MPK6 activate CAMTA3 nuclear export and destabilization (Jiang et al., 2020). Similarly, NAC TF, upon interaction with Ca^2+^/CaM, positively regulates various biotic stress responses in *Solanum lycopersicum* (Wang G. et al., 2016). NAC is also responsive to *Colletotrichum gloeosporioides* and *Ralstonia solanacearum* infection in woodland strawberry (Zhang et al., 2018). WRKY is another Ca^2+^/CaM-dependent TF (Park et al., 2005; Yan et al., 2018) implicated in pathogen incursion (Park et al., 2005; Bai et al., 2018). *WRKY7*, *WRKY45*, *WRKY43*, *WRKY53*, and *WRKY50* in a Ca^2+^-driven manner bind to various isoforms of CaM (Park et al., 2005; Popescu et al., 2007). MYB TF is also well characterized as a Ca^2+^-dependent TF. MYB functions upstream in a vast majority of defense-responsive and abiotic stress-receptive genes (Stracke et al., 2001; Chezem et al., 2017; Li et al., 2019). Similarly taking CMLs into consideration, *AtCML9* works as positive regulator of plant immune response. It was found to be induced by *Pseudomonas syringae* and phytohormones including abscisic acid (ABA) and salicylic acid (SA) (Magnan et al., 2008; Leba et al., 2012). Further, *AtCML9* interacts with WRKY53 and TGA3 TFs, both of which are known to mediate biotic stress responses (Popescu et al., 2007). In concurrence, *AtCML37* and *AtCML42* are associated with defense against herbivorous insects (*Spodoptera littoralis*) (Vadassery et al., 2012; Scholz et al., 2014). Very recently, 17 *AcoCPK* genes from *Ananas comosus* (pineapple) were analyzed for their effect under biotic stress. *AcoCPK1*, *AcoCPK3*, and *AcoCPK6* were shown to render susceptible disease resistance in *A. thaliana* against *Sclerotinia sclerotiorum* (Zhang et al., 2020). Another class of Ca^2+^ sensors, CBLs, are known to specifically interact with a family of plant-specific CBL-interacting protein kinases (CIPKs). CBL interacts with Ca^2+^ and binds with CIPK, resulting in kinase activation. The CBL–CIPK complex actively regulates downstream target proteins by phosphorylation (reviewed by Ma et al., 2020; Tang et al., 2020).

**FIGURE 2:**
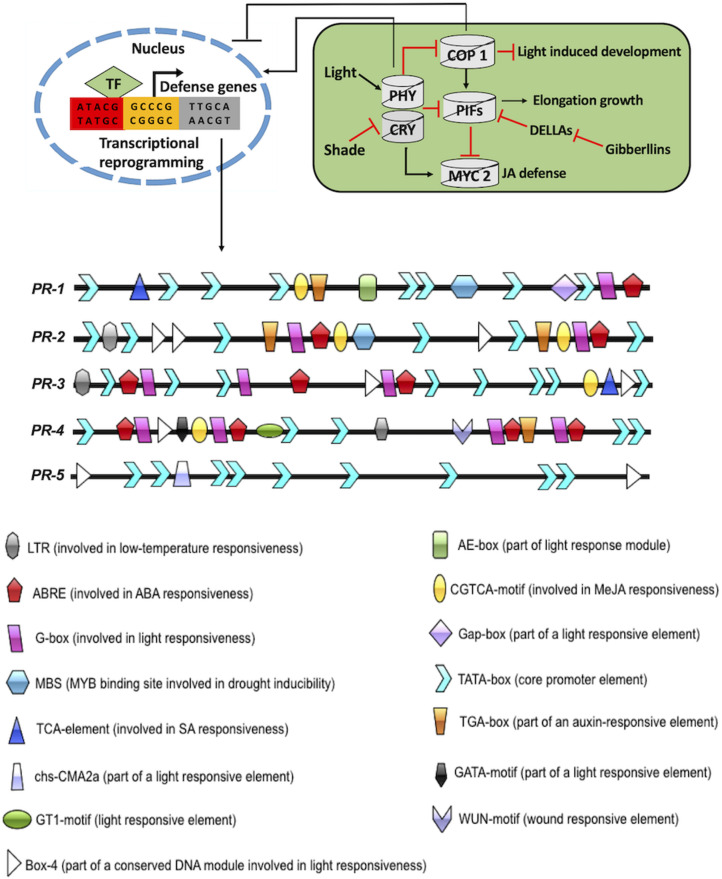
Intersection of plant defense and light signaling. The 1,000-bp upstream sequence of *PR-1*, *PR-2*, *PR-3*, *PR-4*, and *PR-5* were fetched from TAIR10 (https://www.arabidopsis.org/). The motif analysis was done by PLACE database (https://www.dna.affrc.go.jp/PLACE/?action=newplace) and PlantCARE database (http://bioinformatics.psb.ugent.be/webtools/plantcare/html/). The motif structures were drawn using Illustrator for Biological sequences software (http://ibs.biocuckoo.org/).

The other initial responses of pathogen attack on plants include the generation of reactive oxygen species (ROS) and activation of mitogen-activated protein kinases (MAPKs) (Muthamilarasan and Prasad, 2013). ROS and MAPKs overlap with other signaling pathways, including light pathways (Goldsmith and Bell-Pedersen, 2013; Foyer, 2018). Furthermore, pest attack on plants activates local or systemic defense responses involving oligogalacturonoids (OGAs), jasmonic acid (JA), and hydrogen peroxide (H_2_O_2_) signaling pathways (Fürstenberg-Hägg et al., 2013). Plants are also capable of producing volatile compounds that repel attacking pests (discussed in section “Effect of Dark/Light on Plant–Pathogen Interaction and Associated Mechanisms”). These compounds are part of lipoxygenase (LOX) and terpenoid signaling pathways (Pichersky and Gershenzon, 2002; Dudareva et al., 2006). Another pivotal downstream defense mechanism by plants include the generation of defensive proteins and universal stress proteins. These proteins comprise protein inhibitors, lectins, chitinases, α-amylase inhibitors, and polyphenol oxidases (Fürstenberg-Hägg et al., 2013; Lee et al., 2019). Additionally, the role of *pathogenesis-related* (*PR*) genes in plant defense responses has been considerably explored (Ali et al., 2018). *PR* genes translate into proteins that are induced in plants only upon pathological or similar conditions (conditions of non-pathogenic origin) (Jain and Khurana, 2018). They are considered as an important component of plant innate immune response and are implicated in HR and SAR responses (Jain and Khurana, 2018). PR proteins are grouped into 17 families, depending upon their biochemical and molecular properties (van Loon et al., 2006). In *A. thaliana*, five *PR* genes (*PR-1*, *PR-2*, *PR-3*, *PR-4*, and *PR-5*) are routinely explored for their involvement in plant biotic interactions (Hamamouch et al., 2011). *PR-1*, *PR-2*, and *PR-5* are implicated in SA-dependent SAR response, while *PR-3* and *PR-4* are involved in JA-dependent SAR response (Thomma et al., 1998; Hamamouch et al., 2011). An important aspect associated with PR proteins is their simultaneous indulgence in biotic and abiotic stress (Ali et al., 2018). To substantiate this, the 1,000-bp upstream region of all five *PR* genes from *A. thaliana* were analyzed bioinformatically to determine the presence of different motifs associated with a variety of environmental stresses. Intriguingly, all the *PR* genes contained multiple light-responsive motifs (AE-box, GAP-box, GT-1 motif, G-box, GATA-motif, box-4, and chs-CMA2a). The presence of light-responsive motifs in the promoter region of *PR* genes probably implies the binding of light-dependent genes to these conserved sequences (Figure 2). This notion itself supports the idea of intense cross-talks between biotic stress responses and light signaling pathways.

Finally, the involvement of phytohormones in regulating plant biotic defense responses cannot be ruled out. ETI and PTI induces specific downstream signaling pathways, in which three phytohormones are crucial, namely, SA, JA, and ethylene (ET). SA regulatory pathways are responsive to biotrophic and hemi-biotrophic pathogenic agents. Similarly, JA and ET pathways are responsive to necrotrophic agents and chewing pests (Bari and Jones, 2009; De Vleesschauwer et al., 2014). SA stimulates the SAR pathway promoting the expression of *PR* genes, which in-turn renders tolerance against a wide range of pathogens (Grant and Lamb, 2006; Fu and Dong, 2013; Ádám et al., 2018). SA, JA, and ET regulatory pathways for plant defense exhibit significant divergence, but they overlap to render defense against pathogenic agents (Glazebrook, 2005; Ku et al., 2018). Additionally, ABA, auxin, brassinosteroids (BRs), cytokinin (CK), gibberellic acid (GA), and peptide hormones also have vital significance in regulating the immune responses of the plants (Bari and Jones, 2009; Ku et al., 2018; Islam et al., 2019; Chen et al., 2020). Amongst all the phytohormones, JA is critical in triggering the plant defense system and cross-talks with other phytohormonal pathways to stimulate the plant immune responses (Yang et al., 2019).

## Light as an Environmental Cue

Plants are exposed to variable light intensities that encompass light perception and signaling pathways responsible for growth, development, and immune responses (Hua, 2013; Ballaré, 2014). Nevertheless, plants often confront light intensities that exceed their photosynthetic capacity, inducing light stress (Mishra et al., 2012). Mechanisms encompassing light/dark alteration under stress conditions have been comprehensively studied (Mittler, 2002; Cerdán and Chory, 2003; Jiao et al., 2007; Koussevitzky et al., 2007; Mühlenbock et al., 2008; Alabadí and Blázquez, 2009; Chory, 2010; Kami et al., 2010; Lau and Deng, 2010; Trotta et al., 2014; Kaiserli et al., 2015; Saijo and Loo, 2020). Given the extreme importance of light for survival, immunity, growth, and development, plants have evolved the capability to sense and respond to different spectra of light (visible, infrared, ultraviolet, etc.) through photoreceptors. In *A. thaliana*, five distinctive genes (*PHYA–PHYE*) encode phytochrome protein (Clack et al., 1994; Li et al., 2011). They potentially act as receptors for red and far-red lights (Takano et al., 2009). Similarly, in *A. thaliana*, cryptochromes encoded by *CRY1* and *CRY2* dedicatedly sense blue (∼400 nm) and green (500–600 nm) lights and UV-A (Folta and Maruhnich, 2007; Jiao et al., 2007; Bae and Choi, 2008; Jenkins, 2009).

As previously discussed, plants undergo skotomorphogenesis in the absence of light while photomorphogenesis in the presence of light (see section “Introduction”). Repressor proteins such as constitutive photomorphogenic/de-etiolated1/fusca (COP/DET/FUS) inhibit photomorphogenesis under dark conditions (Hardtke and Deng, 2000; Dong et al., 2014). Mutants with defects in any of these repressor proteins display constitutive photomorphogenic (COP) phenotypes under dark conditions (Lau and Deng, 2012). The repressor proteins are characterized into four categories with overlapping functions and have been studied extensively (Deepika et al., 2020; Pham et al., 2020). The first one is COP1, which is a RING-finger-type ubiquitin E3 ligase (Deng et al., 1992). Under dark conditions, it acts as a repressor of light signaling and accumulates in the nucleus (Xu D. et al., 2014). On the contrary, COP1 is exported out of the nucleus, facilitating photomorphogenesis under light conditions (von Arnim et al., 1997; Hardtke et al., 2000; Seo et al., 2003; Duek et al., 2004; Xu et al., 2016a; Podolec and Ulm, 2018). COP1 acts as a central repressor and facilitates ubiquitination and degradation of various positive regulators of light, namely, long hypocotyl in far-red 1 (HFR1), long hypocotyl 5 (HY5), and long after far-red light 1 (LAF1) (Hardtke et al., 2000; Osterlund et al., 2000; Jang et al., 2005; Yang et al., 2005). The degradation of positive regulators of light by COP1 is constrained under light by prohibiting COP1 protein from the nucleus. This triggers the initiation event of photomorphogenesis. The function of COP1 has been extensively linked to light signaling (Figure 2). However, it is also implicated in the regulation of flowering time, circadian rhythm, and temperature signaling (Ma et al., 2002; Yu et al., 2008; Jeong et al., 2010; Catalá et al., 2011; Menon et al., 2016; Wang W.-X. et al., 2016; Xu et al., 2016b; Hoecker, 2017). COP1 is also known to interact with the suppressor of PHYA 1–4 (SPA 1–4). This interaction results in tetrameric complexes comprising two COP1 and two SPA proteins (COP1/SPA complex) (Zhu et al., 2008). SPA proteins are reported to positively enhance COP1 function (Ordoñez-Herrera et al., 2015). Skotomorphogenesis is accomplished by suppressing the expression of genes involved in photomorphogenic development in the dark (Josse and Halliday, 2008). This is tightly regulated by the COP1–SPA1E3 ligase complex (Osterlund et al., 2000; Josse and Halliday, 2008; Ordoñez-Herrera et al., 2015; Holtkotte et al., 2016; Paik et al., 2019). COP1–SPA1E3 ligase targets HY5 TF for degradation by the proteasome (Osterlund et al., 2000). COP1–SPA complex interacts with CULLIN4 (CUL4) to form CUL4–COP1–SPA complex. CUL4–COP1–SPA complex acts as CULLIN ring E3 ligase (CRL) and degrades positively acting TFs under dark conditions to suppress photomorphogenesis (Chen et al., 2010). Interestingly, CUL4–COP1–SPA complex has a dual function in dark/light-induced photomorphogenesis (Zhu et al., 2015; Paik et al., 2019). CUL4–COP1–SPA complex activates early ubiquitin-mediated degradation of phytochrome interacting factor 1 (PIF1) to trigger light-induced seed germination (Zhu et al., 2015; Paik et al., 2019). The second group of repressor protein is COP9 signalosome (CSN). It is highly conserved and comprises eight subunits (Serino and Deng, 2003). CSN had been reported to be implicated in deneddylation/derubylation of CRLs (Schwechheimer et al., 2001). The third group of repressor protein is de-etiolated1 (DET1), COP10, DNA damage-binding protein 1 (DDB1), and CUL4. DET1 is known to bind histone H2B (Benvenuto et al., 2002). It also regulates PIFs and HFRs to suppress seed germination and photomorphogenesis under dark conditions (Dong et al., 2014; Shi et al., 2015). Finally, the fourth group of repressor protein is PIFs (PIF1–PIF8) that belong to basic helix-loop-helix (bHLH) family of TFs and suppresses photomorphogenesis under dark conditions (Leivar et al., 2008; Shin et al., 2009; Leivar and Quail, 2011; Pham et al., 2018). They bind to the G-box consensus sequence in the 1,000-bp upstream region of light-responsive genes. Under dark conditions, phytochromes physically interact with PIFs to repress light response. The activation of photoreceptors suppresses COP1/SPA E3 ubiquitin ligase complexes and PIFs (Martínez et al., 2018b). This eventually activates HY5 to modulate the expression of light-inducible genes and disrupts PIF function (Chen et al., 2013; Toledo-Ortiz et al., 2014; Gangappa and Kumar, 2017). Upon plant exposure to dark conditions, photoreceptor inactivation enables COP1/SPA- and PIF-mediated disruption of light signaling (Xu X. et al., 2014; Xu et al., 2015b, 2017). This signaling cascade promotes plant growth by involving phyto-hormones (such as BR, auxins, and GA) at the cost of plant immunity (Lozano-Durán and Zipfel, 2015; Martínez et al., 2018b).

Photoreceptors are also responsible to determine the quality of light (R:FR ratios). Upon excitation by R light, phytochromes are transformed into FR light-absorbing state (biologically active “Pfr”). Since red light is absorbed by chlorophyll and carotenoids, its quantity is significantly decreased when penetrating through a dense canopy (Slattery et al., 2017; Walker et al., 2018). Shade-intolerant plants (such as *A. thaliana*) perceive and respond to such conditions by elongating stems and promoting flowering (Fiorucci and Fankhauser, 2017). This is an evolutionary phenomenon developed in plants and is termed shade-avoidance syndrome (SAS). Plants exhibit SAS, which is represented by the elongation of plant parts such as hypocotyls, stems, and petioles (Casal, 2013). Both PHYA and PHYB proteins contribute towards SAS. PHYB restrains SAS under R-enriched light (R:FR > 1), while PHYA restrains SAS under FR-enriched light (R:FR < 1) (Franklin, 2008; Lorrain et al., 2008; Franklin and Quail, 2010; Jaillais and Chory, 2010; Martinez-Garcia et al., 2010; Stamm and Kumar, 2010). This also result in the inactivation of PIF to promote BR and auxin production (Martínez et al., 2018a).

The amalgamation of photochemical and non-photochemical processes (NPQ) dissipates excess excitation energy (EEE) of plants as heat. Photochemical- and NPQ-dissipated EEE maintenance is facilitated by the acidification of the chloroplast lumen, involving PSII-associated proteins (Niyogi, 2000; Müller et al., 2001; Li et al., 2004; Niyogi et al., 2005; Ciszak et al., 2015). EEE eventually results in the formation of ROS, H_2_O_2_, superoxide (${\text{O}}_{2}^{.-}$), and singlet oxygen (^1^O_2_), which overlaps with biotic stress signaling. Light/dark alterations induce plant resistance to pathogen infection and oxidative damage in systemic tissues. This indicates a cross-wired signaling between dark/light conditions and biotic stress (Rossel et al., 2007; Mühlenbock et al., 2008; Szechyńska-Hebda et al., 2010; Zhao et al., 2014). EEE induces SAR and basal response to pathogenic biotrophic bacteria. This response alters ROS and redox signals and thus induces SA, ET, and glutathione (Mühlenbock et al., 2008; Szechyńska-Hebda et al., 2010).

## Effect of Dark/Light on Plant–Pathogen Interaction and Associated Mechanisms

Accumulating evidences indicate that plant response to biotic stress cannot be fully deciphered by studying discrete stress response (Suzuki et al., 2014; Dworak et al., 2016). Such notions support comprehensive study in connection with plant responses to simultaneously appearing stresses. Both qualitative and quantitative changes occur in the intensity of light during dark/light alterations. The majority of invertebrate herbivores with few exceptions (Kreuger and Potter, 2001; VanLaerhoven et al., 2003) are more active at night in comparison with day because of parasitism or predation constraints during the day (Hassell and Southwood, 1978). The emission of volatiles also affects herbivory with respect to diurnal variation. There are even qualitative and quantitative disparities during day/night in wound-induced volatiles (De Moraes et al., 2001; Gouinguené and Turlings, 2002; Martin et al., 2003). Taking into account the effect of dark/light on pathogen attack upon plants, the number of airborne fungal spores is significantly high at night (dark) in comparison with day (Schmale and Bergstrom, 2004; Gilbert and Reynolds, 2005; Zhang et al., 2005). On the contrary, few fungal spores peak at day (light) time (Gadoury et al., 1998; Su et al., 2000). Along with dark/light alterations, the plant–pathogen interaction is also influenced by an array of factors such as temperature fluctuations, humidity changes, and leaf surface water content resulting from dew conditions at night (Meijer and Leuchtmann, 2000; Koh et al., 2003). The presence of light also reduces germ tube growth and spore germination in plant pathogenic fungi (Mueller and Buck, 2003; Beyer et al., 2004). A number of studies have revealed that pathogen infection is influenced by light/dark conditions before inoculation happens. Tolerance to aphid infestation was also confirmed by high-light pre-exposures in wild-type plants and mutants impaired in protein phosphatase 2A (PP2A) (Rasool et al., 2014). Similarly, inoculation of *Puccinia striiformis* in wheat (*Triticum aestivum*) seedlings was more at low light intensity than dark-grown seedlings (De Vallavieille-Pope et al., 2002). In a few other instances, inoculation irradiances have been found to be inversely proportional to infection (Shafia et al., 2001), indicating a direct impact of dark/light on host tolerance. Recently, nucleotide-binding NLR Rpi-vnt1.1 proteins have been shown to require light for imparting disease resistance against races of the Irish potato famine pathogen *Phytophthora infestans*, which discharge the effector protein AVRvnt1 (Gao et al., 2020). Glycerate 3-kinase (GLYK), which is a nuclear-encoded chloroplast protein, is necessary for the activation of Rpi-vnt1.1. Under light conditions, AVRvnt1 binds to the full-length chloroplast targeted GLYK isoform triggering of Rpi-vnt1.1. However, under the dark scenario, plants generate a shorter truncated GLYK that is devoid of the intact chloroplast transit peptide, thus compromising Rpi-vnt1.1-mediated resistance. The conversion between full-length and short-length GLYK transcripts is governed by light-dependent promoter selection mechanism. In plants that are devoid of Rpi-vnt1.1, the occurrence of AVRvnt1 decreases GLYK accumulation in chloroplasts, hence reducing GLYK contribution to basal immunity. The findings are thus clearly depictive of the fact that the pathogen-driven functional alteration of the chloroplast results in a light-dependent immune response (Gao et al., 2020). Plausibly, plants are more prone to pathogen attack in the dark than during the day. However, it cannot be held true for all pathogens attacking the plant systems.

There occur two mechanisms that contribute to the regulation of plant defense responses during dark/light fluctuations: first, the energetic significance of light-dependent chemical reactions (depends on the capacity of photosynthetic electron transport to produce ATP and reducing power); and second, perception of light (shade and R:FR exposure conditions) and regulation of downstream light-dependent signaling pathways (Roberts and Paul, 2006). The following subsections highlight both the mechanisms with respect to photosynthesis, ROS accumulation, and light signaling.

### Photosynthetic Processes and Reactive Oxygen Species Accumulation in Biotic Stress

**FIGURE 3:**
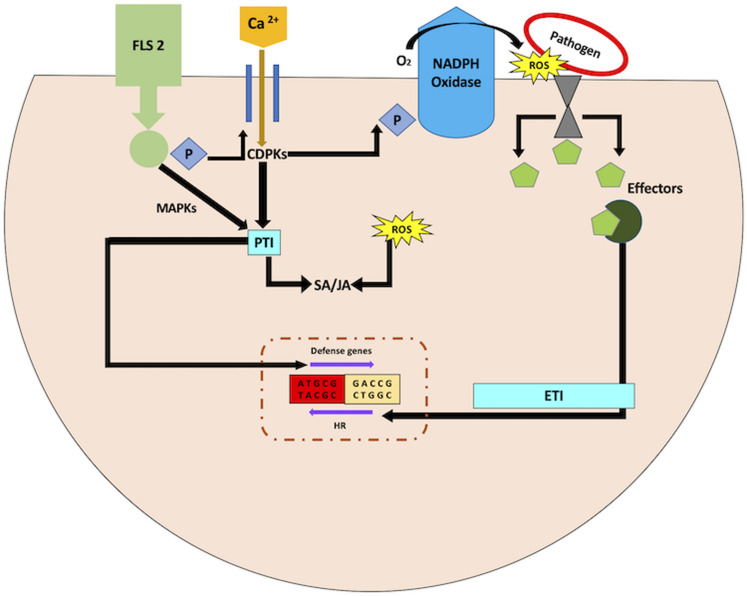
ROS modulation of biotic stress responses. FLS2 receptor kinase triggers the Ca^2+^ flux, followed with mitogen-activated protein kinase (MAPK) and Ca^2+^–dependent protein kinase (CDPK) cascades. These initial signals contribute to pathogen-associated molecular pattern (PAMP)-triggered immunity (PTI). Effector molecules are synthesized by host-adapted microbes, which suppress PTI. Plant system under such circumstances specifically identifies effector molecules to activate effector-triggered immunity (ETI). This eventually initiates the hypersensitive response (HR).

Photosynthesis captures light energy via electron transport chain (ETC) for assimilation of carbon dioxide as well as repair and growth of plant body. The vital metabolites so produced from photosynthesis are utilized in carbon fixation, fatty acid biosynthesis, assimilation of nitrogen into amino acids, etc. (Nunes-Nesi et al., 2010). These light-driven pathways occurring in chloroplast can impact short term-induced plant defense responses (Delprato et al., 2015). Intriguingly, some part of the biosynthetic pathways of ABA, JA, and SA (plant defense hormones) also occur in the plastids (Bobik and Burch-Smith, 2015). This might impact plant defense in the dark due to the hormonal cross-talk in plant–microbe interaction. Moreover, chloroplast acts as a site for ROS generation upon stress perception. Leaves get acclimatized to light fluctuations during growth and development, as calvin cycle enzymes and light-harvesting complexes are adjusted to efficiently manage the available light. However, photosynthetic electron transport produces more electrons when carbon fixation is halted or light fluctuations occur. This helps in the generation of more electrons for the electron acceptor NADP^+^. Under such circumstances, free electrons from ETC are transferred to oxygen leading to ROS generation. Additionally, the light-dependent events and pathways occurring in the chloroplast impact short and long-term-induced plant defense responses via photorespiration resulting in the generation of H_2_O_2_ in the peroxisomes (Lu and Yao, 2018). Under acute light stress conditions, impairment in chlorophyll synthesis and disruption of chloroplast can also lead to the accumulation of ROS. This might surpass the potential of the antioxidant system in the chloroplast (Apel and Hirt, 2004). Nevertheless, ROS has also been very well implicated in plant defense against pathogens (Torres, 2010; Nath et al., 2017; Huang et al., 2019), and any deviation of the redox balance in the chloroplast can impact ROS regulated plant defense (Figure 3). For instance, lipid peroxidation occurs when ROS accumulates upon biotic stress perception (De Dios Alché, 2019). The repercussions of the requisite of light/dark fluctuations for chloroplast-derived ROS goes far beyond direct signaling functions of ROS.

As for post pathogen attack, some of the products of lipid peroxidation are reactive electrophiles with a carbonyl group (Vollenweider et al., 2000). These electrophiles are a consequence of ROS impact on membrane lipids or are products arising from lipoxygenase enzyme activity. Many amongst these electrophiles are imperative signaling molecules implicated in the regulation of cell death and defense gene expression (Vollenweider et al., 2000; Alméras et al., 2003; Thoma et al., 2003; Cacas et al., 2005). Hence, light/dark fluctuations impact the production of ROS-derived electrophiles. Such cases are reported in interactions amongst plants and pathogens or their elicitors. Taking into consideration the response of cryptogein (a known elicitor), cell death was mediated by ROS accumulation in light conditions (Montillet et al., 2005). On the contrary, when plants are subjected to dark conditions, cell death is independent of ROS accumulation and correlates with specific lipoxygenase activity (Montillet et al., 2005).

The primary source of ROS during biotic stress response is not the chloroplast. It is rather NADPH oxidase (respiratory burst oxidase) that is localized in the plasma membrane (Apel and Hirt, 2004; Figure 3). This implies that chloroplast-derived ROS in the presence of light may not help with pathogen defense. Nonetheless, this may or may not hold true, since NADPH oxidase does not impede the production of chloroplast-derived ROS. More so, lesion mimic mutants with random necrotic lesions are characterized to comprehend the underlying mechanisms involved in signaling of biotic stress tolerance (Lorrain et al., 2003). These necrotic lesions on the leaves are comparable with those generated in response to HR. Lesion mimic mutants have higher expression of *PR* genes and enhanced resistance against pathogen attack. These mutants highlight the common nexus between biotic stress response and chloroplast ROS based on two observations (Karpinski et al., 2003; Bechtold et al., 2005). First, the formation of lesions in lesion mimic mutants are light-dependent (Brodersen et al., 2002). Second, the functional characterization of these mutants highlights genes implicated in chlorophyll biosynthesis or degradation (Ishikawa et al., 2001; Mach et al., 2001; Pružinská et al., 2003; Wang F. et al., 2016; Lv et al., 2019). Additionally, the change in expression profiles of genes implicated in chlorophyll biosynthesis also leads to light-dependent lesion mimic phenotypes, eventually resulting in enhanced disease tolerance (Molina et al., 1999; Lv et al., 2019). This may be due to the formation of ROS generated by the effect of light on chlorophyll intermediates acting as photosensitizers. The electrons are excited by the absorption of light energy by photosensitizers. The ROS thus produced acts as signals for pathogen resistance responses. Hence, it is evident that light-derived ROS from either free photosensitive pigments or photosynthetic light-harvesting complexes can influence plant defense signaling.

**FIGURE 4:**
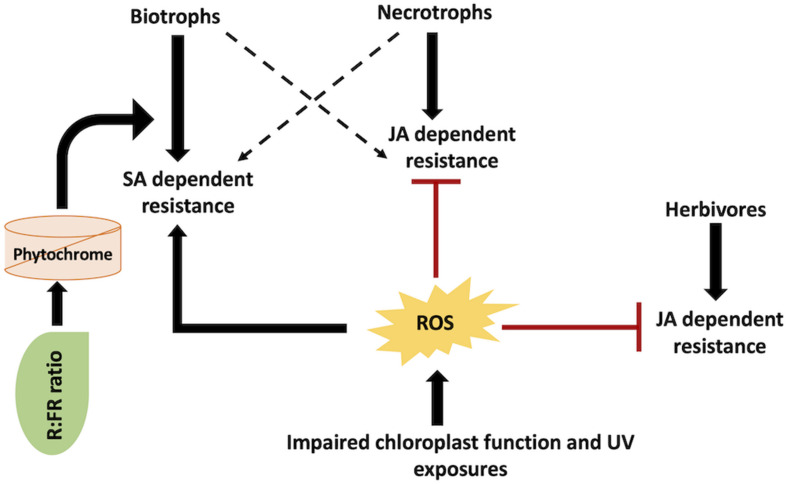
SA/JA mediated cross-talk in light signaling and defense responses against different pathogenic agents: various biotic agents activate different mechanisms. Reactive oxygen species (ROS), salicylic acid (SA), and jasmonic acid (JA).

Plants have decentralized well-defined mechanisms for light-derived ROS in tissues subjected to biotic stress. For instance, the *A. thaliana chlorophyllase 1* (*AtCHL1*) gene is implicated in chlorophyll degradation and removal of photosensitive porphyrin ring intermediates. *AtCHL1* functions to preclude ROS accumulation due to damaged chloroplast (Kariola et al., 2005). This particular gene has been established to be triggered upon necrotrophic infections (Kariola et al., 2005). Plants with impaired *AtCHL1* gene display enhanced tolerance to *Erwinia carotovora* (necrotrophic bacterial pathogen) but reduced tolerance to *Alternaria brassicicola* (a fungal necrotroph) (Kariola et al., 2005). SA-dependent pathway is involved in *E. carotovora* resistance, while JA-dependent pathway is involved in *A. brassicicola* resistance. SA- and JA-mediated plant defense responses are antagonistic in nature (Figure 4). As such, *AtCHL1* mediates the equilibrium between SA- and JA-dependent plant–pathogen resistance pathways by adjusting ROS accumulation from chlorophyll metabolites. Similarly, the *A. thaliana ACD2* gene decreases the accumulation of photosensitizers. This results in an increased resistance to *P. syringae* (Mach et al., 2001). It is also noteworthy that several plants generate photosensitizers, which directly play a prominent role in imparting biotic stress tolerance. Phototoxins produce ROS in the presence of white or UV light that directly prevents herbivore or pathogen infection (Downum, 1992; Flors and Nonell, 2006). On the contrary, few fungal pathogens themselves generate photosensitive toxins (namely, cercosporin) leading to plant cell necrosis (Daub and Ehrenshaft, 2000). An entire range of various levels of interaction amongst light, dark, and biotic stress constitutes induced defenses in plants. These levels of interaction include ROS generation, phytochrome signaling, and activation of biotic stress-related genes. Taken together, different biotic agents deploy overlapping signaling pathways with ROS as the key modulator molecule (Figure 4). Thus, comprehending the significance and pathways involved in these overlapping responses may be useful in deciphering the overall involvement of light/dark alterations on biotic stress tolerance and resistance mechanisms.

### Perception of Light With Respect to Shade and R:FR Exposures; and Regulation of Downstream Light-Dependent Signaling Pathways

The second key mechanism by which light/dark alterations regulate biotic stress responses engages direct light-responsive signaling pathways. The Genoud et al. (1998) has elegantly unraveled this mechanism in *A. thaliana*. The group has identified *psi2* light signaling defective mutant that develops light-dependent random necrotic lesions and has an increased expression of *PR1* gene (Genoud et al., 1998). Further characterization of *psi2* mutant reveals that the biotic stress responses are governed by light at various levels. For example, *PSI2* regulates the responses associated with phytochrome. Moreover, PHYA and PHYB are essential for *PR* gene expression and light-dependent HR lesion formation (Genoud et al., 1998, 2002). Hence, the phytochrome mutants have decreased resistance to *P. syringae*, while the *psi2* mutants have enhanced resistance to *P. syringae.* This is a clear evidence where light signals play a pivotal role in the regulation of induced biotic resistance. However, why and how phytochrome signaling modulate biotic stress responses still remain obscure. On the contrary, the dark conditions or high light stress also operates molecular pathways that are common with pathogen responses (Mackerness et al., 1999; Rossel et al., 2002; Izaguirre et al., 2003; Kimura et al., 2003; Stratmann, 2003; Zhao et al., 2014). Enormous literature exists on the physiological basis of light dependency in relation to biotic defense, but the in-depth basis of dark/light effect on induced resistance remains elusive. A vital question, therefore, is to ascertain the mechanistic details for such observations. Undoubtedly, light is indispensable for plant growth and development, meaning that there is no unambiguous explanation to connect various observations across distinctive scales of organization. However, there are two modules that can be taken into account: first, resistance, which decreases the rigor of pathogen attack by restricting the activity of pathogen; and second, tolerance, which decreases the adverse effects of pathogen attack on the host plant. The demarcation between resistance and tolerance is critical for comprehension of interaction mechanisms between dark/light and plant defense.

**FIGURE 5:**
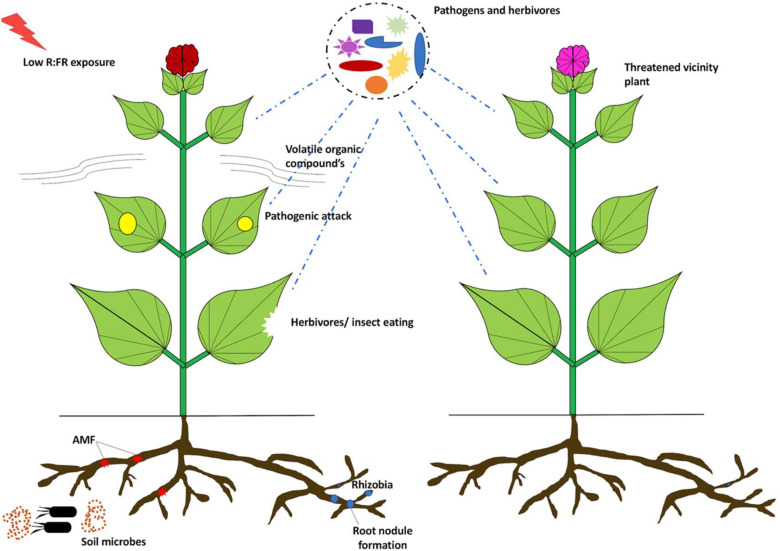
Low R:FR modulation of plant immunity: low R:FR makes the plants more susceptible to pathogens and insects. Low R:FR exposure modulates VOC compositions, exposing plants to herbivory attack. Also, the formation of nodules and arbuscules is impacted by R:FR ratios. Arbuscular mycorrhizal fungi (AMF), volatile organic compounds (VOCs).

In the field, the shade affects the cumulative radiation balance with plausible influence on biotic environment of the host. The temperature of the surrounding air and organisms is usually lower in shade, influencing a wide range of biological processes including biotic stress. For instance, tree canopies influence the species richness of insectivorous birds that affects herbivory (Strong et al., 2000; Van Bael and Brawn, 2005; Nell et al., 2018). Similarly, canopy shade has varying effects on photosynthetically active radiation (PAR) and UV wavelength (Grant and Heisler, 2001; Heisler et al., 2003; Grant et al., 2005). Additionally, the shade also results in either infestation by many pathogens or protection from the others. Pathogenic infestation is more stern in shade, for example, anthracnose (*C. gloeosporioides*) of *Euonymus fortunei* (Ningen et al., 2005), powdery mildew (*Microsphaera alphitoides*) on oak (*Quercus petraea*) (Kelly, 2002), and coffee rust (*Hemileia vastatrix*) (Soto-Pinto et al., 2002). Nonetheless, very often, plants develop a symbiotic relationship with beneficial microbes to enhance their defense responses and obtain nutrients under deficit conditions. The intense interplay between light signaling and defense mechanisms against beneficial and harmful microorganisms might be imperative for plant growth on high planting densities. Taking into account the beneficial interactions, the best-studied example is the nitrogen-fixing rhizobium bacteria and the leguminous plants (Ferguson et al., 2010). Rhizobium colonizes plant roots to form nodules that fix atmospheric nitrogen into mineral nitrogen for efficient usage by the leguminous plants. In return, the bacteria get carbon sources from the plant, which is essential for their survival (Ferguson et al., 2010). The *Lotus japonicus PhyB* mutant displays a shade-avoidance phenotype (similar to *Arabidopsis* mutant) with lesser number of root nodules in contrast to control plants (Suzuki et al., 2011; Sessa et al., 2018). Experimental validation reveals that the nodulation is decreased in grafted plants with *phyB* shoots and control roots. This is indicative of the fact that the mutations in the shoot tissue decrease nodulation in the roots (Suzuki et al., 2011; Shigeyama et al., 2012). The decreased nodulation in *phyB* mutants can be linked to downregulation of JA-responsive gene expression leading to lower JA levels in roots (Suzuki et al., 2011; Shigeyama et al., 2012). Next, taking into account the impact of R:FR exposures, the plant defense mechanisms against herbivores and pathogens are downregulated under low R:FR conditions (Ballaré, 2014; Ballaré and Austin, 2019; Figure 5). This probably implies that the interplay between beneficial interactions and light signaling is species-specific. In yet another example, plants establish a symbiotic relationship with arbuscular mycorrhizal fungi (AMF). These phosphate-acquiring fungi form “arbuscules” to enable phosphate and nitrogen uptake in plants, and in return, they derive carbon sources from plants (Keymer et al., 2017). The exposure of low R:FR ratios to *L. japonicus* roots decreases hyphal development of the AMF *Rhizophagus irregularis*. This is tightly regulated by the downregulation of JA-responsive genes resulting in decreased JA levels in root exudates (Nagata et al., 2015, 2016). At high plant density area, symbiotic relationship with rhizobium and AMF may be under scrutiny during low R:FR light conditions. However, the relationship between plant–microbe beneficial interactions and light signaling is still unclear and requires further investigation to improve plant growth and immunity.

Plants possess a continuous ever-evolving armor of defense mechanisms to prevent the colonization of harmful pathogens (Jones and Dangl, 2006; Nishad et al., 2020). Plants identify the signatures from the impeding pathogens and microbes via PAMPs, HAMPs, ETI, and PTI (Zipfel, 2014; Cui et al., 2015; Peng et al., 2018) (see section “Biotic Stress and Plant Defense Responses”). As already discussed, the antagonistic relationship between JA and SA modulates defense responses against biotrophic and necrotrophic pathogens (Glazebrook, 2005). JA is the central regulatory phytohormone coordinating the defense responses against pathogens and insects (Turner et al., 2002; Santino et al., 2013; Yang et al., 2019). Initial studies indicated that plants exposed to low R:FR or with impaired *PHYB* gene function exhibit reduced resistance to herbivores that is associated with declined sensitivity to JA (McGuire and Agrawal, 2005; Izaguirre et al., 2006; Moreno et al., 2009). Upon herbivory attack, volatile organic compound (VOC) emissions and methyl jasmonate (MeJA)-associated gene expression decreases in *A. thaliana* under low R:FR exposures (Kegge et al., 2013; Figure 5). A similar observation has been reported in barley where low R:FR exposure modifies constitutive VOC emissions to regulate the responses associated with plant–plant interactions (Kegge et al., 2015). This is further confirmed in *Solanum* (Cortés et al., 2016). In tomato, a low R:FR ratio affects MeJA-mediated VOC composition. This in turn influences the indirect defense response by enticing the insects (Cortés et al., 2016). Additionally, an intricate regulation of light signaling pathways maintains a balance of the constructive or destructive effects of light on plant growth and immunity. In contrast to the above observations, under low R:FR conditions, *Geranium robertianum* (a shade adapted forest understory plant) does not display downregulation of its JA-related plant defenses (Gommers et al., 2017). It also exhibits a slight increase in resistance against *B. cinerea*. Transcriptome analysis of *G. robertianum* and *Geranium pyrenaicum* (a shade-avoiding plant) reveals a number of genes with an opposite mode of regulation upon encountering shade conditions. Under low R:FR conditions, receptors like kinases FER and THE1 (responsible for shade induced elongation growth) are induced in *G. pyrenaicum*. FER and THE1 may be directly involved in regulating plant immunity and growth under shade. Conversely, in *G. robertianum*, exposure to low R:FR ratios leads to suppression of *JAZ* genes, which confer immunity under shade conditions. This establishes a classical example of the plasticity of light signaling in modulating plant growth and defense responses (Hématy et al., 2007; Kessler et al., 2010; Stegmann et al., 2017). Phenotypic and transcriptomic studies unravel a link between SAS- and SA-based defense components in shade-unresponsive *Arabidopsis* mutants (Nozue et al., 2018). JA, SA, and auxin-related signaling pathways are stimulated under low R:FR conditions and contribute strongly toward SAS (Nozue et al., 2018). Prolonged photoperiods positively regulate SA production, SA-related defenses, systemic immunity, and autoimmunity in lesion-mimic mutants (Griebel and Zeier, 2008; Gangappa and Kumar, 2017). Shade-avoidance mechanism under low light conditions restrains defense via a number of mechanisms (Cipollini, 2004). The swing in the distribution of resources to growth under shade may compete with the allocation of resources to plant defense. There might also be an intersection between light signaling and defense signaling. Under shade, stem elongation is regulated by auxin and gibberellins (Vandenbussche and Van Der Straeten, 2004). Auxin is known to interact with defense signaling pathways via a cross-wired mesh involving indole acetic acid (IAA). IAA also decreases JA-regulated generation of defense compounds (Baldwin et al., 1997; Yang et al., 2019). Contrariwise, the expression and concentration of auxins are altered upon wounding and herbivory (Cheong et al., 2002; Schmelz et al., 2003; Machado et al., 2016). Even the stiffening of the cell wall is an antagonistic mechanism between plant defense and shade (Cipollini, 2004), where gibberellin causes cell wall loosening resulting in cell expansion in shade. This can be attributed as an imperative component of plant defense.

Extensive research has been devoted to the mechanistic details as to how phytochromes regulate JA responses in relation to biotic defense responses (Hou et al., 2010; Ballaré, 2014; Leone et al., 2014; Pieterse et al., 2014; Campos et al., 2016). The described mechanism involves the interaction between DELLA proteins (growth repressor) and JAZ proteins (negative defense regulator) (Ballaré, 2014; Pieterse et al., 2014). MYC2 has been very well implicated to activate downstream defense responses (Hou et al., 2010; Verhage et al., 2012; Woldemariam et al., 2013; Liu et al., 2019). The DELLA proteins are degraded to sequester JAZ, resulting in inhibition of MYC2 TF (Hou et al., 2010). JAZ10 protein has been observed to be highly stable in *A. thaliana phyB* mutant. This could probably be due to the degradation associated with DELLA proteins (Leone et al., 2014). Again, the lower sensitivity of the *jaz10 phyB* double mutant than the *phyB* mutant to *B. cinerea* highlights the importance of JAZ10 in relation to light signaling and biotic stress responses (Cerrudo et al., 2017). Particularly, inactivation of *PHYB* suppresses JA-related plant defense responses exclusive of shade-avoiding morphological changes (Moreno et al., 2009). In contrast, the JAZ absence reinforces JA-related plant defenses without compromising plant growth in *phyB* (Campos et al., 2016). Thus, plant defense activation or suppression is not dependent upon growth promotion or inhibition. This is suggestive of the fact that growth, light signaling, and defense trade-off are effective adaptive responses. Both JA- and SA-dependent defense responses are downregulated under low R:FR conditions. This also overlaps with NPR1 phosphorylation inhibition leading to reduced defense induction (de Wit et al., 2013). Also, for JA-related defense responses, prolonged photoperiods require the involvement of PHYA, cryptochromes, DELLAs, and the JA-regulating TF MYC2 (Cagnola et al., 2018). Conversely, short photoperiods result in PIF4-mediated growth elevation and immunity suppression. This is in concert with the fact that the elevated PIF4 accumulation and activation in the dark are dependent upon COP1/DET1 (Gangappa et al., 2017; Gangappa and Kumar, 2017). The COP1/DET1–PIF4 complex is also essential for autoimmunity suppression at high temperatures in *snc1* and *cpr5* mutants (Gangappa and Kumar, 2017). These studies are indicative of crucial involvement of the COP1/DET1-PIF module in prioritizing growth over plant immunity.

In addition, BR signaling apart from being involved in growth responses also plays a vital role in biotic stress responses (Planas-Riverola et al., 2019). BR signaling is linked with flagellin (a well-known PAMP) recognition upon pathogen attack. This is accomplished by the interaction between the BR receptor kinase BRI1 and its coreceptor BAK1 (Chinchilla et al., 2007). BR inhibits the defense machinery of plants by inducing *Brassinazole-resistant 1* (*BZR1*) gene (Lozano-Durán et al., 2013; Lozano-Durán and Zipfel, 2015). BZR1 is an important component of the BAP/D module, which is very well implicated in plant growth and development (Bouré et al., 2019). Under low R:FR conditions, BR responses may be involved in growth via the BAP/D module that can supersede flagellin-mediated plant defense response. It is also pertinent to mention that low R:FR affects the primary metabolism of plants (Yang et al., 2016; de Wit et al., 2018). Upon infecting plants, pathogens target carbohydrates as the key source of carbon for their survival. The enhanced susceptibility under low R:FR or in the phytochrome mutants may be due to higher accessibility of carbohydrates by the pathogens in plant tissues. Secondary metabolite production and defense-related gene expression (*viz.* MAPK and *PR* genes) are usually correlated with high concentrations of sugar accumulation in plant tissues (Bolouri Moghaddam and Van Den Ende, 2012). Reduced plant defense has been observed for *B. cinerea* under low R:FR conditions (Cargnel et al., 2014). This obstructed plant defense is a result of declined defense-related gene expression and metabolite production (Cargnel et al., 2014). Thus, low R:FR exposure declines defense-related pathways and enriches soluble sugars in plants, eventually inducing lesion formation in infected plant tissue (Figure 5). Taken together, plant growth responses to shade conditions are intricately cross-wired with the immune response generated by the plants upon pathogen exposure.

## Conclusion and Future Prospects

Exposure of plants to a combination of adverse environmental cues such as biotic stresses and light fluctuations coerces the efforts to meet enormous food demand. Despite the massive usage of pesticides and insecticides in the last few decades, the overall crop losses due to pathogen attack have not been reduced significantly. Monitoring infection time, plant growth, and other important parameters such as light/dark conditions can result in a better understanding of plant defense toward pathogens, particularly when extrapolated to field conditions. The present review provides an elaborate information on how plants perceive and respond to multiple dark/light alterations and biotic stresses. Light and dark conditions together or independently modulate a diverse range of signaling pathways to control pivotal plant growth and defense regulators. The function of multi-faceted dark/light signaling intermediates such as COP, CRY, PHY, and PIF has been extensively covered to highlight the impact of dark and light modulations on plant biotic defense responses. Even though significant efforts have been made to deep dive into the plant–microbe interactions and their association with light signaling, the mechanistic details encircling this complex intersection are obscure. Thus, the basic research to comprehend the mechanisms involved in the integrated circuitry of plant immunity and dark/light interactions, at both biological and ecological scales, will pave the way to overcome the limitations associated with crop losses globally.

## Author Contributions

MIA conceptualized and designed the study. ZI, MSI, AH, and EFA compiled the data and wrote the manuscript. All authors have read the manuscript and agreed for publication.

## Conflict of Interest

The authors declare that the research was conducted in the absence of any commercial or financial relationships that could be construed as a potential conflict of interest.
